# A FACS-based novel isolation technique identifies heterogeneous CTCs in oral squamous cell carcinoma

**DOI:** 10.3389/fonc.2024.1269211

**Published:** 2024-02-26

**Authors:** Anshika Chauhan, Arnab Pal, Meenakshi Sachdeva, Geeta S. Boora, Monil Parsana, Jaimanti Bakshi, Roshan Kumar Verma, Radhika Srinivasan, Debajyoti Chatterjee, Arindam Maitra, Sushmita Ghoshal

**Affiliations:** ^1^ Department of Biochemistry, Post Graduate Institute of Medical Education and Research, Chandigarh, India; ^2^ Department of Regenerative Medicine, Post Graduate Institute of Medical Education and Research, Chandigarh, India; ^3^ Department of Otolaryngology, Post Graduate Institute of Medical Education and Research, Chandigarh, India; ^4^ Department of Cytology and Gynecological Pathology, Post Graduate Institute of Medical Education and Research, Chandigarh, India; ^5^ Department of Histopathology, Post Graduate Institute of Medical Education and Research, Chandigarh, India; ^6^ National Institute of Biomedical Genomics, Kalyani, West Bengal, India; ^7^ Department of Radiotherapy, Post Graduate Institute of Medical Education and Research, Chandigarh, India

**Keywords:** circulating tumour cells, oral squamous cell carcinoma, fluorescence-activated cell sorting (FACS), phenotypic heterogeneity, whole transcriptome amplification, qRT-PCR

## Abstract

**Purpose:**

Isolating circulating tumour cells (CTCs) from the blood is challenging due to their low abundance and heterogeneity. Limitations of conventional CTC detection methods highlight the need for improved strategies to detect and isolate CTCs. Currently, the Food and Drug Administration (FDA)-approved CellSearch™ and other RUO techniques are not available in India. Therefore, we wanted to develop a flexible CTC detection/isolation technique that addresses the limitation(s) of currently available techniques and is suitable for various downstream applications.

**Methods:**

We developed a novel, efficient, user-friendly CTC isolation strategy combining density gradient centrifugation and immuno-magnetic hematogenous cell depletion with fluorescence-activated cell sorting (FACS)-based positive selection using multiple CTC-specific cell-surface markers. For FACS, a stringent gating strategy was optimised to exclude debris and doublets by side scatter/forward scatter (SSC/FSC) discriminator, remove dead cells by 4′,6-diamidino-2-phenylindole (DAPI) staining, and eliminate non-specific fluorescence using a “dump” channel. APC-labelled anti-CD45mAB was used to gate remaining hematogenous cells, while multiple epithelial markers (EpCAM, EGFR, and Pan-Cytokeratin) and an epithelial–mesenchymal transition (EMT) marker (Vimentin) labelled with fluorescein isothiocyanate (FITC) were used to sort cancer cells. The technique was initially developed by spiking Cal 27 cancer cells into the blood of healthy donors and then validated in 95 biopsy-proven oral squamous cell carcinoma (OSCC) patients. CTCs isolated from patients were reconfirmed by Giemsa staining, immuno-staining, and whole transcriptome amplification (WTA), followed by qRT-PCR. *In vitro* culture and RNA sequencing (RNA-Seq) were also performed to confirm their suitability for various downstream applications.

**Results:**

The mean detection efficiency for the Cal 27 tongue cancer cells spiked in the whole blood of healthy donors was 32.82% ± 12.71%. While ~75% of our patients (71/95) had detectable CTCs, the CTC positivity was independent of the TNM staging. The isolated potential cancer cells from OSCC patients were heterogeneous in size. They expressed different CTC-specific markers in various combinations as identified by qRT-PCR after WTA in different patients. Isolated CTCs were also found to be suitable for downstream applications like short-term CTC culture and RNA-Seq.

**Conclusion:**

We developed a sensitive, specific, flexible, and affordable CTC detection/isolation technique, which is scalable to larger patient cohorts, provides a snapshot of CTC heterogeneity, isolates live CTCs ready for downstream molecular analysis, and, most importantly, is suitable for developing countries.

## Introduction

In solid cancers, cancer cells disseminate from the primary tumour, intravasate, and finally reach circulation. These cancer cells, along with the other host cells and components of the microenvironment milieu, diversify, adapt over time through clonal evolution, and play an important role in the metastatic process. These epithelial cancer cells identified from the peripheral blood are termed circulating tumour cells (CTCs). Moreover, various studies have shown that CTCs act as a precursor for metastasis and, in recent years, emerged as a promising tool for liquid biopsy to diagnose micro-metastatic disease as well as to monitor and predict the course of solid cancers. CTCs are present in the blood even in the very early stages of the disease, much before clinical confirmation of metastases (1). However, the detection and isolation of CTCs are challenging, being present in a very low proportion against a massive background of other cells in the blood. Most approaches exploit differential cell surface marker expression and/or distinct physical and molecular characteristics of CTCs from other cells present in the blood (2). Being phenotypically heterogeneous, the specifically chosen marker may not be expressed by all CTCs leading to bias in the isolation strategy. For example, EpCAM is a widely used marker for detecting CTCs, but this poses a major limitation, as EpCAM is the marker for well-differentiated epithelial cells (3). It is one of the first downregulated makers during the epithelial–mesenchymal transition (EMT) process, making the identification of CTCs with mesenchymal transformation difficult (4, 5). To address this problem, various groups isolate CTCs by negative selection, i.e., depleting normal blood cells significantly using antibodies specific to cell surface molecules on blood cells, e.g., CD45 (6). However, the major limitation of the negative selection strategy is that the purity of isolated CTCs is compromised, and using those samples for downstream molecular analysis may lead to erroneous conclusions. Physical property-based approaches also possess major disadvantages; i.e., these properties are highly variable among CTCs with considerable overlap with those of other non-cancerous blood cells. The diameter of CTCs is inconsistent and ranges from 6 µm to more than 20 µm, while most blood cells have a diameter of approximately 10 µm (7). Thus, size-based approaches that exploit the bigger CTCs with respect to haematogenous cells for their isolation might miss the isolation of small-sized CTCs (8), which we have documented in our previous study on oral squamous cell carcinoma (OSCC) patients (9). Studies have shown the prognostic role of CTCs in head and neck squamous cell carcinoma (HNSCC) including OSCC (10, 11).

We designed an efficient, user-friendly fluorescence-activated cell sorting (FACS)-based CTC isolation strategy considering the various limitations of available CTC isolation strategies. Our technique is scalable to a large cohort of patients without needing any specialised equipment or technical expertise. This approach combines pre-enrichment by immuno-magnetic hematogenous cell depletion followed by FACS-based positive selection of CTCs using multiple cell-surface markers labelled with a single fluorochrome. It was optimised using Cal 27, a tongue cancer cell line, spiked in the blood from the healthy volunteers and analytically validated to isolate a heterogeneous population of CTCs without leucocyte contamination; thus, it is ready to use in various downstream molecular analyses.

## Methods

### Cell lines

The human OSCC cell line Cal 27 (CRL-2095™, ATCC^®^) was used in spike-in experiments, and HEK293T (CRL-3216™, ATCC^®^) cells were used for generating puromycin resistance gene expressing stable Cal 27 cell line. Cell lines were authenticated using short tandem repeat profiling and confirmed mycoplasma-free. Cell lines were maintained at 37°C, 5% CO_2_ in Dulbecco’s modified Eagle’s medium (DMEM) media (#12-604F, Lonza, Basel, Switzerland) supplemented with 10% foetal bovine serum (#10270106, Gibco, Grand Island, NY, USA) and Antibiotic-Antimycotic (#15240062, Gibco). Cultured cells were passaged once they reached 70%–80% confluency. All subsequent spike-in experiments were performed at least 48 h after passaging.

### Characterisation of Cal 27 cell line

The expression of various markers that were used for CTC isolation and reconfirmation was analysed in Cal 27 cell line (EpCAM, EGFR, CK, and Vimentin) using various techniques like flow cytometry, immunofluorescence, and qRT-PCR. Giemsa staining was performed to characterise the morphology of cells.

#### Flow cytometry

Cal 27 cells were trypsinised, washed, and resuspended in phosphate-buffered saline (PBS) upon reaching 70%–80% confluency. Whole blood (1 mL) was diluted in 1:2 dilution by PBS. Trypsinised cancer cells were spiked in diluted blood. Approximately 2 mL of diluted blood was carefully layered over 1 mL of HiSep™ LSM 1077 (#LS001, HiMedia, Mumbai, India) in a 15-mL conical tube and centrifuged at 400 g for 30 min at 22°C in a swinging bucket rotor. The upper layer was aspirated without disturbing the mononuclear cell layer at interphase. The mononuclear cell layer was carefully transferred to the other conical tube. Then, PBS was added to this tube, mixed, and centrifuged at 250 g for 10 min at 4°C. The supernatant was discarded.

For cell counting, 10 µL of cell suspension was mixed with an equal volume of trypan blue dye, and cells were placed on a haemocytometer and counted under an inverted microscope. Approximately 100,000 cells (Cal 27 and peripheral blood mononuclear cells (PBMCs) separately) were resuspended in PBS–ethylenediaminetetraacetic acid (EDTA) and subjected to staining for 1 h at 4°C using the following antibodies:

1. EpCAM Monoclonal Antibody (VU-1D9), fluorescein isothiocyanate (FITC) (#MA1-10197, Invitrogen, Carlsbad, CA, USA),
2. Pan-Cytokeratin Monoclonal Antibody (C-11), FITC (#MA5-28561, Invitrogen),
3. EGFR Monoclonal Antibody (ICR10), FITC (#MA5-28104, Invitrogen),
4. Vimentin Monoclonal Antibody (V9), FITC (#11-9897-80, Invitrogen),
5. CD45 Monoclonal Antibody, APC (#IM2473U, Beckman Coulter, Brea, CA, USA), and
6. 4′,6-Diamidino-2-phenylindole (DAPI) (#D3571, Invitrogen™).

Cells were washed, resuspended with PBS–EDTA, and then analysed using a flow cytometer (Navios, Beckman Coulter; BD FACS Aria™ III) after employing a range of internal quality controls. Data were analysed using Kaluza version 2.1 (Beckman Coulter).

#### Immunofluorescence

Cal 27 cells were fixed with neutral buffered saline for 60 min and washed with PBS. Fixed cells were blocked with bovine serum albumin in PBS for 30 min at 4°C and incubated with all CTC-specific antibodies (EpCAM, EGFR, Pan CK, and Vimentin) and DAPI for 1 h. The excess antibody was washed out with PBS. Stained cells were placed on poly-l-lysine-coated slides, air-dried, and observed under a fluorescence microscope.

#### qRT-PCR experiments

Total RNA was isolated from Cal 27 cells using QIAzol^®^ lysis reagent (#79306, Qiagen, Valencia, CA, USA) followed by cDNA synthesis using QuantiTect Reverse Transcription Kit (#205311, Qiagen) following the manufacturer’s protocol. qRT-PCR was run using specific primers following SYBR green chemistry on CFX96™ Real-Time System (Bio-Rad, Hercules, CA, USA).

#### Giemsa staining

Suspension of cells was placed on poly-l-lysine-coated slides and air-dried. Slides were fixed with methanol for 20 min and subjected to Giemsa staining (1:30 diluted in double-distilled water) for 30 min. Stained cells were observed under an inverted microscope for cancer cell-specific morphological characteristics.

### Cell spike-in experiments

#### Cell preparation for spike-in

All subsequent spike-in experiments were performed at least 48 h after passaging. Cells were trypsinised, washed, and resuspended in PBS upon reaching 70%–80% confluency. For cell counting, 10 μL of cell suspension was mixed with an equal volume of trypan blue dye, and cells were placed on a haemocytometer and counted under an inverted microscope. For each experiment, cells were counted in triplicates.

#### Recruitment of healthy donors

Five healthy donors were recruited for spiking experiments after obtaining their written consent as per the Institute Ethics Committee’s approval (Approval no. NK/5989/PhD/183). Approximately 5 mL of whole blood from healthy donors was collected in an EDTA vial (Becton Dickinson, Franklin Lakes, NJ, USA).

**Figure 1:**
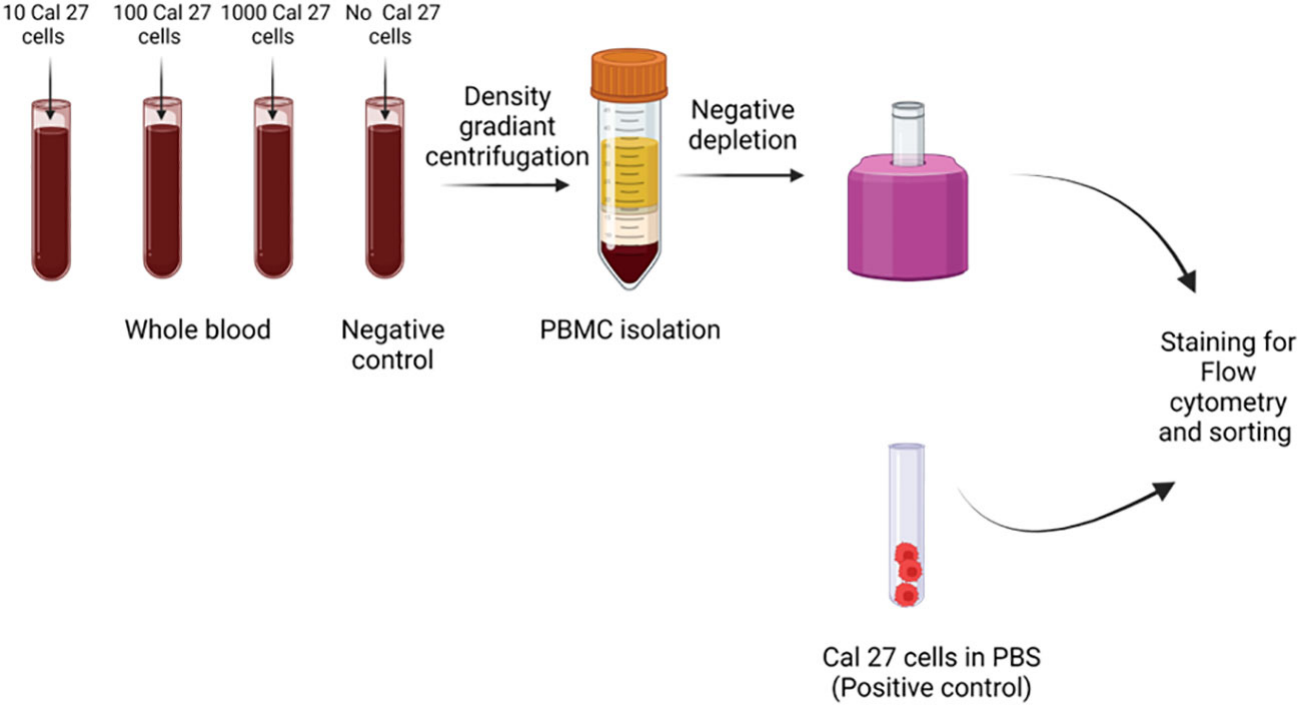
Cell spiking experiments were performed by adding different numbers of Cal 27 tongue cancer cells (10, 100, and 1,000/mL) into the whole blood of healthy donors. Then, samples were subjected to peripheral blood mononuclear cell (PBMC) isolation, followed by CD45+ cell depletion, and finally, detection/isolation using fluorescence-activated cell sorting (FACS). Along with positive control (only Cal 27 cells), a negative control (only whole blood without any Cal 27 cells added) was processed as a sample.

Cells were spiked in the blood of five different healthy donors in different concentrations of 10, 100, and 1,000 cells in 1 mL of whole blood in triplicates for each concentration. As a negative control, 1 mL of blood was processed with the same protocol without adding Cal 27 cells. As a positive control, Cal 27 cells stained with all antibodies were also processed (**Figure 1**).

#### PBMC isolation

PBMCs were isolated using the protocol mentioned above. PBMCs were resuspended in 0.5 mL PBS containing 2% foetal bovine serum (FBS) and 1 mM EDTA.

#### Negative depletion

The isolated PBMCs were transferred to a 5-mL round-bottom tube and subjected to negative depletion using EasySep™ Human CD45 Depletion Kit II (#17898, Stemcell Technologies, Vancouver, BC, Canada) following the manufacturer’s protocol. This CD45+-depleted cell suspension was centrifuged at 400 g for 5 min at 4°C to settle the CD45− cells. Initially, PBMCs showed slight positivity when stained with CTC-specific antibodies. A blocking step was added post-negative depletion to reduce the non-specific signal. CD45-depleted cell suspension was blocked using a blocking buffer (10% human serum, 2% bovine serum albumin (BSA), and 2 mM EDTA in PBS) for 45 min.

#### Positive selection of CTCs by FACS

CD45− cells enriched by EasySep™ Human CD45 Depletion Kit II were incubated by the above-described FITC-fluorochrome-conjugated CTC-specific monoclonal antibodies, APC-conjugated CD45 monoclonal antibody, and DAPI. Excess antibodies were washed out, and cells were sorted on a FACS using the standardised gating strategy for isolating pure and viable CTCs. All APC-negative and FITC bright, positive cells were sorted in a tube.

#### Gating strategy for isolation of CTCs by FACS

**Figure 2:**
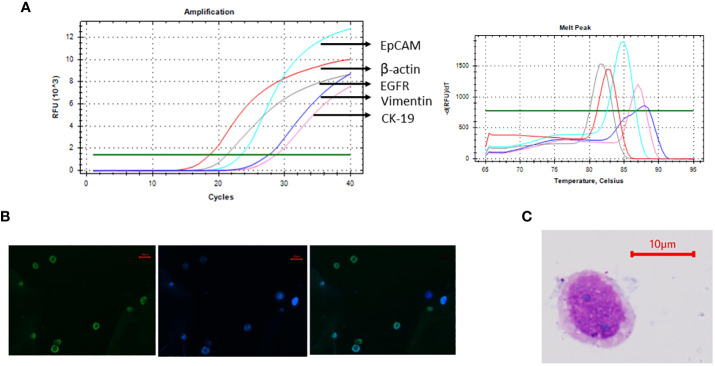
Cal 27 cell line was characterised using various methods. **(A)** mRNA expression analysis of Cal 27 cells by qRT-PCR showed that Cal 27 cells express all circulating tumour cell (CTC)-specific markers (EpCAM, EGFR, CK-19, and Vimentin). **(B)** Immunofluorescence microphotographs showing bright fluorescein isothiocyanate (FITC) fluorescence in Cal 27 cells upon incubation with an antibody mixture of all four CTC-specific markers. **(C)** Giemsa staining of Cal 27 cells showed characteristic cancer cell-like morphology with high nuclear-to-cytoplasmic ratio and coarse chromatin.

A FACS gating strategy was optimised for sorting pure CTCs out of the huge background of PBMCs (**Figure 2**). The formula calculated detection efficiency: Number of cells detected in FACS/Number of cells initially spiked * 100. A negative control tube (only blood without cancer cell spiked was processed) was run at every run, and gating was set accordingly.

### Characterisation of sorted cells

Sorted cells were reconfirmed by Giemsa staining (morphological characterisation), immunocytochemistry (phenotypical characterisation), and qRT-PCR for CTC-specific markers (molecular characterisation).

#### Immunocytochemistry

Immunocytochemistry was performed using BenchMark XT autoimmunostainer (Ventana Medical Systems, Inc., Tucson, AZ, USA) with appropriate positive and negative controls run concurrently. Harvested cells were plated onto coated slides, and blocking was performed using blocking serum. After incubation with blocking serum, sections were incubated using a primary antibody, i.e., cytokeratin cocktail (AE1 and AE3, #313M-16, Sigma, St. Louis, MO, USA) with dilution 1:300, followed by a horseradish peroxidase-conjugated secondary antibody. Lastly, the antibody-antigen complex was detected using 3,3′-diaminobenzidine. Finally, the slide was stained with haematoxylin for nuclear staining. Brown membrane/cytoplasmic staining was considered positive.

#### qRT-PCR

Sorted cells were subjected to whole transcriptome amplification using REPLI-g WTA Single Cell Kit (#150063, Qiagen) following the manufacturer’s protocol. This kit amplifies the whole transcriptome of the cells ranging from 1 to 1,000, allowing the gene expression analysis at ultra-low cell level. Amplified cDNA was analysed using qRT-PCR. Using specific primers, CTC-specific markers EpCAM, EGFR, CK-19, and Vimentin, and hematopoietic marker CD45 were detected. β-Actin/18S rRNA was used as an internal control.

We utilised three qRT-PCR strategies for the reconfirmation of sorted CTCs (*cDNA = 1:100 diluted whole transcriptome amplification (WTA) product).

- 10 μL SYBR reaction using 4 μL cDNA*,
- 25 μL SYBR reaction using 10 μL cDNA*, and
- 10 μL TaqMan Assays 4 μL cDNA*.

The primer sequence and qRT-PCR conditions are shown in the **Supplementary Material**.

### Experiments to check the specificity of the technique

For checking the specificity of the strategy, a modified Cal 27 cell line was used for spike-in experiments.

Cal 27 cells were transduced with the pLenti CMV puro Vector (#17448, Addgene, Cambridge, MA, USA) inserted with *CRNN* gene. This vector contains the puromycin resistance gene as a selection marker. Transduced cells were then selected with puromycin (5 µg/mL) (#P8833, Sigma) for 7–10 days. The detailed protocol is provided in the **Supplementary Material**.

The modified Cal 27 cell line was spiked into whole blood in different concentrations of 10 cells/mL, 100 cells/mL, and 1,000 cells/mL. These spiked samples were processed for standardised CTC isolation protocol (PBMC isolation → CD45 depletion → sorting of cells using CTC isolation gating strategy). The sorted cells were subjected to whole transcriptome amplification using REPLI-g WTA Single Cell Kit (#150063, Qiagen) followed by qRT-PCR using SYBR green chemistry, and mRNA expression of the puromycin resistance gene was detected.

### Isolation and downstream applications of CTCs isolated from OSCC patients

#### Patients

The present study was approved by the Institutional Ethics Committee of the Post Graduate Institute of Medical Education and Research (NK/5989/PhD/183). Ninety-five patients with histologically confirmed OSCC undergoing surgery were enrolled in the study. Approximately 10 mL of whole blood was collected in an EDTA vial (Becton Dickinson, Franklin Lakes, NJ, USA) after obtaining written informed consent to participate in the present study before surgery. None of our enrolled patients received any form of anticancer treatment before sampling.

#### CTC isolation and characterisation

PBMCs were isolated from the whole blood of the patients, followed by negative depletion by using EasySep™ Human CD45 Depletion Kit II (Stemcell Technologies). CD45-depleted cell suspension was blocked using blocking buffer (10% human serum, 2% BSA, and 2 mM EDTA in PBS) for 45 min and stained for CTC-specific and CD45 monoclonal antibodies for 1 h, followed by washing with PBS. CTCs were sorted by FACS using the optimised gating strategy. To confirm CTC positivity, sorted cells were subjected to whole transcriptome amplification using REPLI-g WTA single cell kit (#150063, Qiagen) as per manufacturer protocol followed by qRT-PCR for control gene (β-actin/18S rRNA), CTC-specific genes (EpCAM, EGFR, CK, and Vimentin), and CD45. The samples positive for the control gene and any combination of CTC-specific markers and negative for CD45 were considered CTC-positive samples. Isolated CTCs from patients were also characterised by Giemsa staining, immunofluorescence, and immunocytochemistry, as described above.

#### Short-term CTC culture

CTCs isolated from OSCC patients were sorted in DMEM:F12 (#BE04-687F/U1, Lonza™) culture media containing insulin (20 μg/mL), epidermal growth factor (20 ng/mL), fibroblast growth factor (10 ng/mL), and 1× Antibiotic/Antimycotic. The cell suspension was plated onto low-adherent 6-well plates and was successfully cultured for up to 4 weeks.

#### Ultra-low cell RNA sequencing of CTCs

Isolated CTCs from 24 OSCC patients were subjected to ultra-low cell RNA sequencing libraries prepared using SMART-Seq^®^ Stranded Kit (#634442, Takara Bio USA, Inc., Mountain View, CA, USA), and paired-end sequencing of 2 × 100 bp was performed on NovaSeq 6000. BCL files were converted to per-read FASTQ using bcl2fastq Conversion Software v2.2. The paired-end reads were quality-checked using FastQC, version 0.11.7. The paired-end reads were aligned to the human reference genome using the aligner STAR (v 2.6) (12). The aligned and sorted bam files were quantified using HTSeq-count (v 0.6.1) (13).

## Results

### Characterisation of the cell line

**Figure 3:**
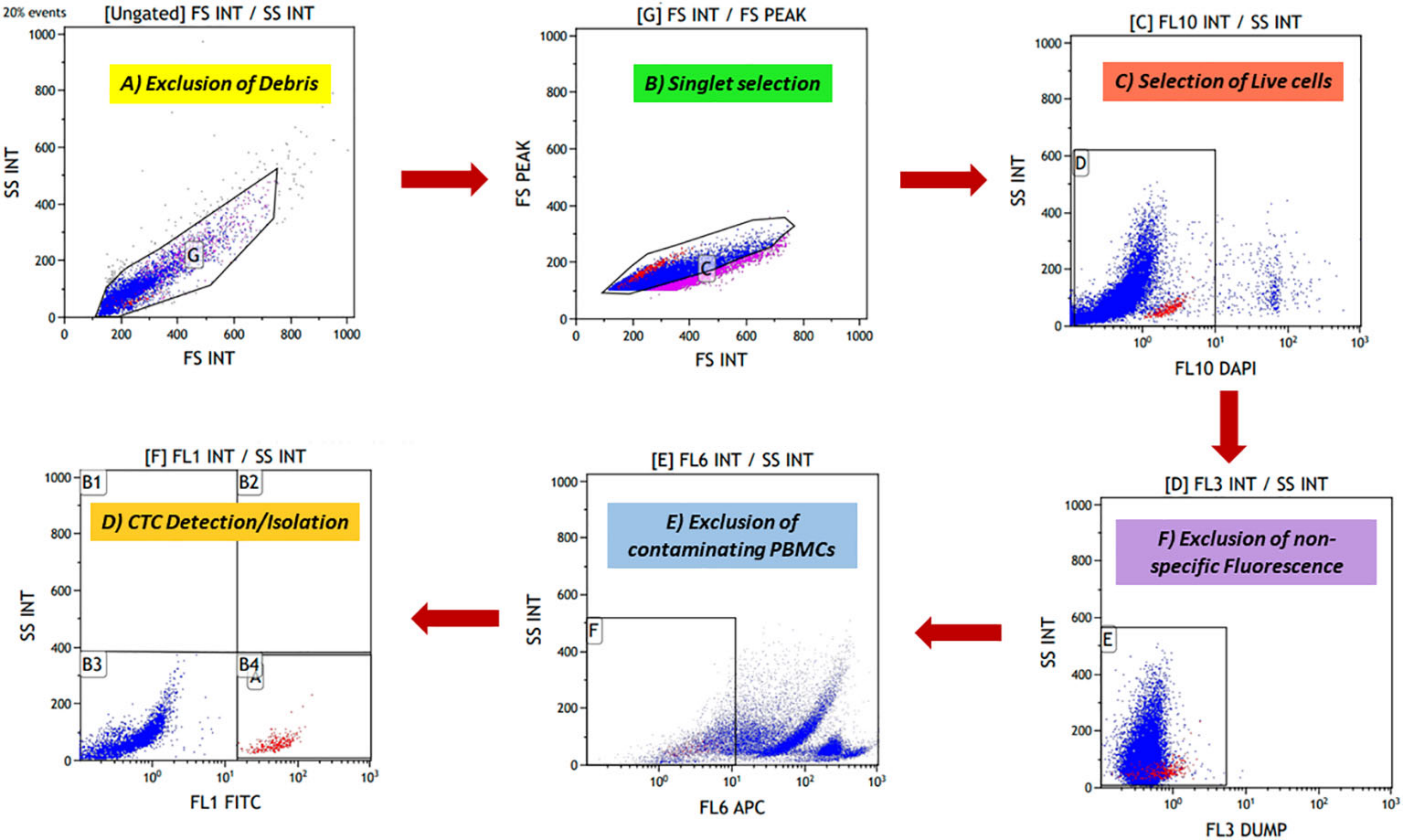
The novel fluorescence-activated cell sorting (FACS) gating strategy for identifying potential cancer cells. **(A)** Forward scatter (FSC) and side scatter (SSC) discriminators were used to exclude debris from cell preparation. **(B)** Doublet events were excluded after debris exclusion, and singlet events were selected using FS peak versus FS integral gate. **(C)** 4′,6-Diamidino-2-phenylindole (DAPI) is a cell-impermeable dye that stains the nucleated cells. DAPI-positive cells were potentially the dead nucleated cells; these cells were excluded. **(D)** A dump channel (Texas red) was included; any conjugate against this channel was not added in cell preparation. Events showing positivity for this channel were considered non-specific events. **(E)** Contaminating CD45+ hematopoietic cells were excluded using the APC channel. **(F)** Finally, fluorescein isothiocyanate (FITC)-labelled potential cancer cells were selected and isolated.

Cal 27 cell line, a tongue squamous cell carcinoma cell line, was used as mock CTCs for optimising the gating strategy and calculating the detecting efficiency of the technique. Before using the cell line for spiking experiments, the cell line was characterised using the markers used for CTC isolation and reconfirmation. Cal 27 cells were positive for all the CTC-specific markers (EpCAM, EGFR, CK-19, and Vimentin) detected using qRT-PCR (**Figure 3A**). Cal 27 cells did not show positivity for CD45 in qRT-PCR (data not shown). Immunofluorescence microphotographs showed bright FITC fluorescence when stained with the cocktail of antibodies for CTC-specific markers (**Figure 3B**). Cal 27 cells showed characteristic cancer cell-like morphology with a high nuclear-to-cytoplasmic ratio and coarse chromatin (**Figure 3C**).

### Novel gating strategy for detecting and isolating heterogeneous CTCs

To isolate rare CTCs from a huge background of blood cells, a highly specific gating strategy was needed to strictly differentiate between cancer cells and blood cells. Various exclusion gates were introduced to ensure the optimal recovery of rare events against a massive number of background events. First, side scatter (SSC) and forward scatter (FSC) discriminators allowed the exclusion of debris from the preparation, followed by FS peak versus FS integral, which allowed the selection of singlet events. Excluding debris and doublet events is crucial, as they can lead to false positivity during rare cell detection. Next, DAPI was added to exclude dead cells, as it is a cell-impermeable dye in low concentrations. DAPI-negative events potentially are viable cells. Another exclusion gate was included, i.e., the “dump” channel (Texas red), as any conjugate is not added for this channel, and events showing positivity for this channel were considered non-specific events. Lastly, the APC channel was added to exclude the remaining CD45+ blood cells. For optimal recovery of cancer cells, three epithelial-specific markers (EpCAM, EGFR, and cytokeratin) and one EMT marker (Vimentin) were added. Using both epithelial and EMT markers in combination labelled with the same fluorochrome, FITC opened the possibility of capturing heterogeneous CTCs effectively without much need for any compensation controls to be run every time, reducing the time and expertise required. Potential CTCs were defined as singlet events with DAPI^−^DUMP^−^CD45^−^FITC^+^ (prototype image of gating strategy in **Figure 2**).

**Figure 4:**
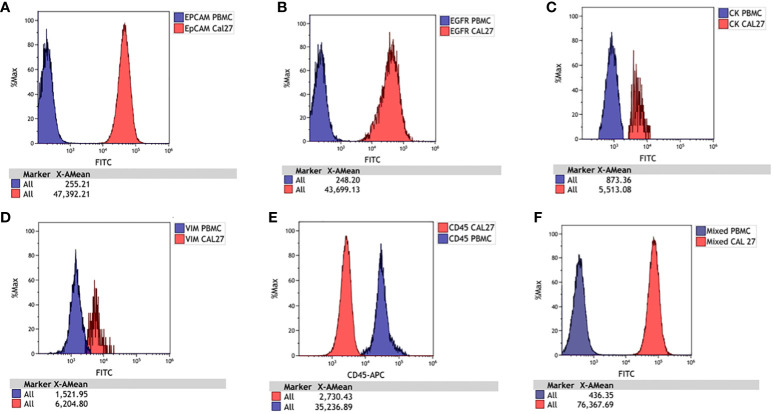
Histograms for Cal 27 and freshly isolated peripheral blood mononuclear cells (PBMCs) stained separately with each antibody and run on a flow cytometer. The signal of PBMCs was denoted with blue, and Cal 27 was denoted with red. The signals of Cal 27 and PBMCs were clearly distinguished using each antibody separately and as a mixed cocktail. Cal 27 and PBMCs stained with **(A)** EpCAM, **(B)** EGFR, **(C)** Pan CK, **(D)** Vimentin, **(E)** CD45, and **(F)** mixed antibody cocktail.

**Figure 5:**
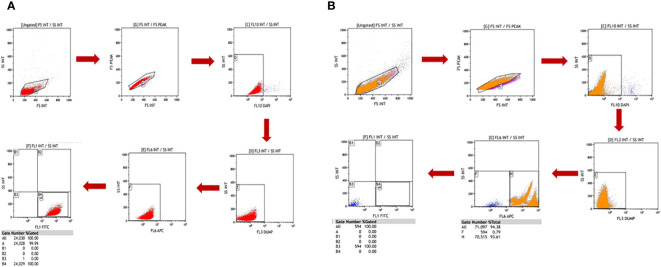
Freshly isolated peripheral blood mononuclear cells (PBMCs) and Cal 27 cells were stained separately with a cocktail of all antibodies (monoclonal antibodies against EpCAM, EGFR, Pan CK, Vimentin, and CD45) and 4′,6-diamidino-2-phenylindole (DAPI) and then detected on a flow cytometer using the standardised gating strategy. The gating strategy distinguished between PBMCs and Cal 27 cells clearly. Gate B4/A, shown in **(A)**, had 100% Cal 27 cells, and Gate B4/A in **(B)** had 0% PBMC. This showed that our strategy clearly distinguished between PBMCs and Cal 27 cells, and events in the B4/A gate were purely cancer cells.

We used Cal 27 cell line and whole blood (for isolating PBMCs) from healthy donors to standardise and finalise the gating strategy. We validated the specificity of antibodies by labelling Cal 27 cells and PBMCs using a single antibody and DAPI, followed by reading on the flow cytometer using the gating strategy. All the antibodies were clearly distinguished between Cal 27 cells and PBMCs using the gating strategy with minimal cross-reactivity (**Figure 4**). We also ran a positive control that contained only Cal 27 cells stained with a cocktail of antibodies (EpCAM, EGFR, Pan CK, and Vimentin) and DAPI. Almost 99.99% of Cal 27 cells showed positivity for the FITC channel, and the PBMC population was completely negative for FITC. In comparison, 93.61% of PBMCs showed positivity in the APC channel using our gating strategy (**Figure 5**). This indicated that the gating strategy was able to stringently discriminate between cancer cells and blood cells.

In conclusion, the stringent and highly specific gating strategy allows strict differentiation between cancer cells and PBMCs.

### Detection efficiency of technique

**Figure 6:**
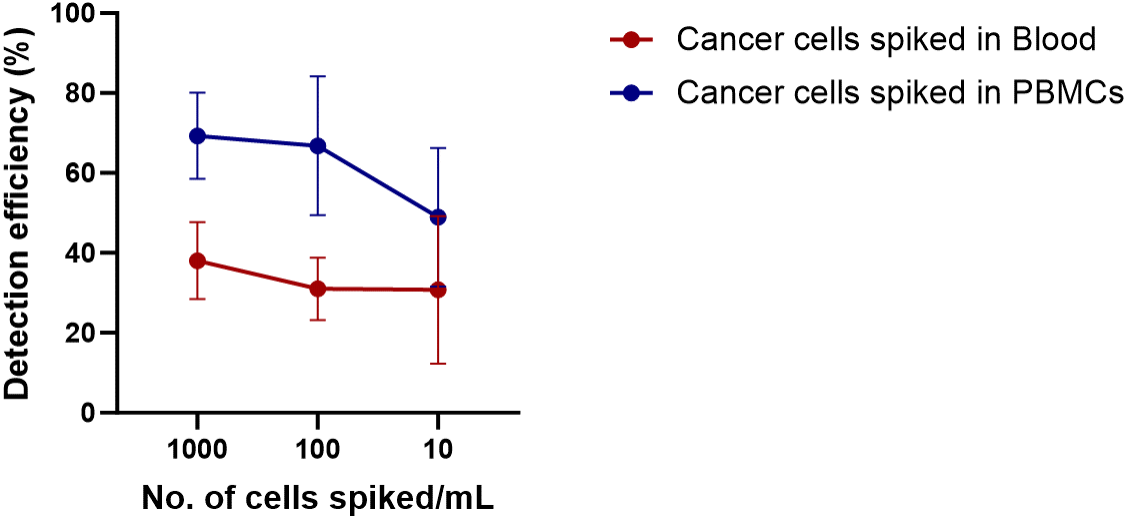
Detection efficiency of the technique was calculated by spiking Cal 27 cells in different number (10 cells/mL, 100 cells/mL, and 1,000 cells/mL) in whole blood or freshly isolated peripheral blood mononuclear cells (PBMCs), followed by CD45+ cell depletion and detection by fluorescence-activated cell sorting (FACS).

Next, to calculate the detection efficiency of the technique, Cal 27 cells in different concentrations were spiked into the blood of five healthy donors by counting using a haemocytometer. Cal 27 spiked blood was subjected to PBMC isolation by density gradient centrifugation followed by CD45 depletion. Finally, CD45-depleted cell suspension was stained with CTC-specific antibodies (EpCAM, EGFR, Pan CK, and Vimentin), CD45, and DAPI and read on the flow cytometer using standardised gating strategy (Cal 27 cell spiking into whole blood → PBMC isolation → CD45 depletion → detection and sorting of cells using CTC isolation gating strategy). A positive control containing only Cal 27 cells stained with all fluorochromes was also processed. A negative control tube (only blood without cancer cell spiked was processed) was run at every run, and gating was set accordingly. Detection efficiency was calculated by the following formula: Number of cells detected in FACS/Number of cells initially spiked * 100. The mean detection efficiency of Cal 27 cells spiked in the whole blood of healthy donors was 32.82% ± 12.71%. For 1,000, 100, and 10 Cal 27 cells spiked per mL of blood, the mean detection efficiency was 38.08% ± 9.62%, 31.04% ± 7.80%, and 30.75% ± 18.45%, respectively (**Figure 6**). There was no significant difference in the detection efficiencies of different numbers of cells spiked.

Adding cancer cells into the whole blood of healthy donors might lead to the destruction of cancer cells by host-immune attack. Therefore, we wanted to calculate the detection efficiency when cancer cells were spiked into isolated PBMCs instead of whole blood (PBMC isolation → Cal 27 cell spiking → negative depletion → detection and sorting of cells using CTC isolation gating strategy). The mean detection efficiency of Cal 27 cells spiked in PBMCs isolated from 1 mL of blood was 63.46% ± 15.79%. The mean detection efficiency when 1,000, 100, and 10 Cal 27 cells were spiked in PBMCs isolated from 1 mL of blood was 68.99% ± 11.24%, 66.82% ± 17.40%, and 53.06% ± 14.14%, respectively (**Figure 6**). No significant difference was observed in detection efficiencies at different numbers of cells spiked in PBMCs.

In conclusion, the detection efficiency of the technique was higher when cancer cells were spiked to PBMCs instead of whole blood. Spiking of cancer cells in whole blood might lead to their killing by immune cells, or loss of cancer cells during density gradient centrifugation might explain these observations.

### Reconfirmation and characterisation of isolated cells

**Figure 7:**
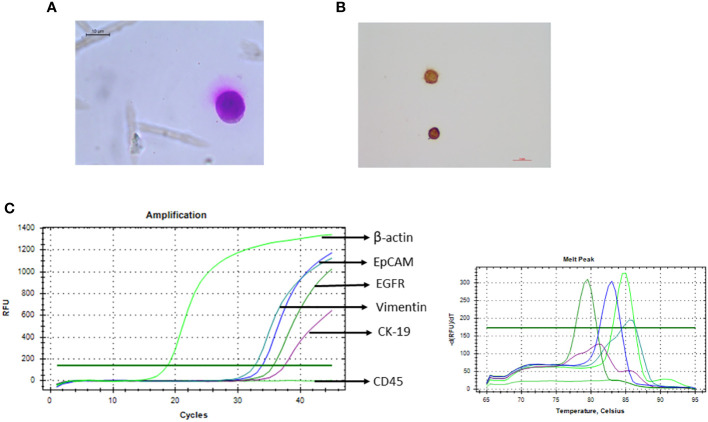
Recovered potential Cal 27 cells, after sorting, were reconfirmed/characterised using various methods. **(A)** Giemsa staining was performed for morphological characterisation like high nuclear-to-cytoplasmic ratio and cancer cell-like morphology. **(B)** Immunocytochemistry microphotograph shows cytokeratin positivity in sorted cells. **(C)** qRT-PCR was performed to analyse the expression of various circulating tumour cell (CTC)-specific genes.

Potential cancer cells were reconfirmed following sorting by various methods like Giemsa staining (morphological characterisation), immunocytochemistry (phenotypical characterisation), and qRT-PCR (molecular characterisation). When subjected to Giemsa staining, sorted cells showed cancer cell-like morphology, i.e., irregular shape with large size (compared to other blood cells) and high nuclear-to-cytoplasmic ratio. No contaminating blood cells were observed on the slide (**Figure 7A**). The sorted cells also showed cytokeratin positivity when analysed by immunocytochemistry (**Figure 7B**).

For molecular characterisation of sorted cells, three qRT-PCR strategies were tried; 25 μL SYBR reaction using 10 μL amplified and diluted cDNA* was observed to be more consistent and sensitive. Because the sorted cell number was very low, we obtained very low Ct values. Therefore, to reduce any possibility of any kind of contamination that can lead to positivity, a positive control (PC-Cal 27 cDNA for CTC-specific genes and PBMC cDNA for CD45) as well as no template control (NTC) was run every time. Sorted cells were positive for all four CTC-specific genes (EpCAM, EGFR, CK-19, and Vimentin) and the control gene (β-actin/18S rRNA) while negative for hematopoietic marker CD 45 (**Figure 7**).

In conclusion, sorted potential cancer cells exhibited morphological, phenotypical, and molecular characteristics of cancer cells, and sorted cell fraction was free of any contaminating CD45+ blood cells.

### Specificity of the technique

**Figure 8:**
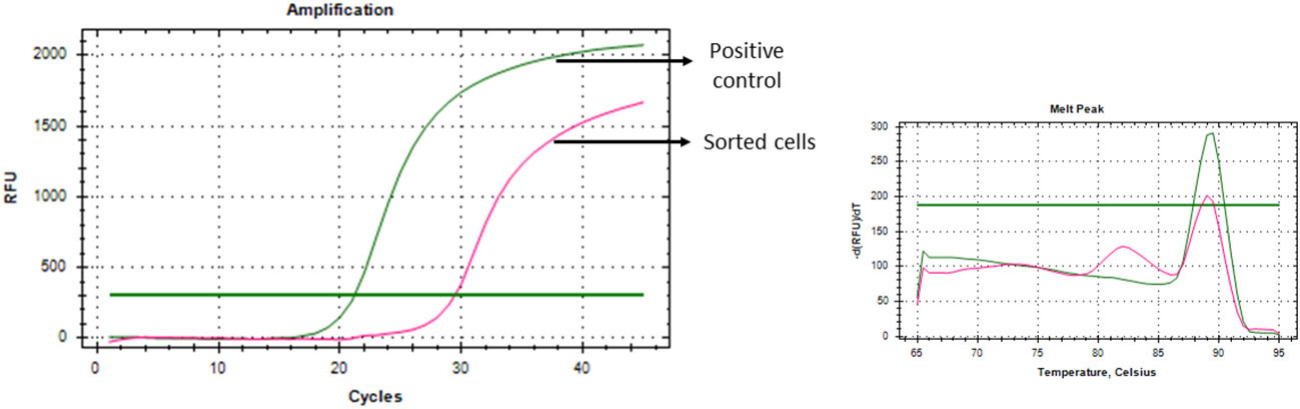
Puromycin resistance gene-expressing Cal 27 cells were spiked into whole blood, isolated using a developed technique, and showed puromycin resistance gene expression as analysed by qRT-PCR, proving the specificity of the developed technique.

To ensure the specificity of the developed CTC isolation technique, as a proof of concept, puromycin resistance gene expressing Cal 27 cells were spiked into the blood of healthy donors (1,000 cells/1 mL of whole blood) and sorted using the gating strategy (Cal 27 cell spiking into whole blood → PBMC isolation → CD45 depletion → detection and sorting of cells using optimised gating strategy). Sorted cells were subjected to whole transcriptome amplification and qRT-PCR for CTC-specific genes and puromycin resistance genes. Interestingly, sorted cells showed positivity for CTC-specific genes and puromycin resistance genes (**Figure 8**). As the puromycin resistance gene is absent in eukaryotic cells, the positivity in qRT-PCR clearly suggested that sorted cells were the initially spiked puromycin resistance gene expressing Cal 27 cells.

### CTCs isolated from OSCC patients revealed phenotypic heterogeneity

**Figure 9:**
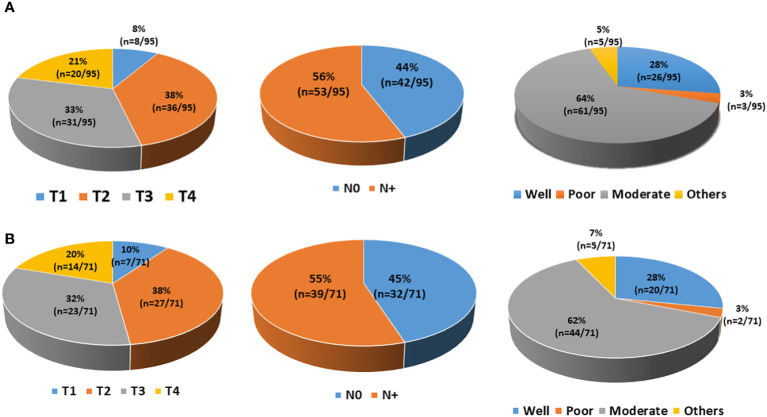
Demographic details of the patients. **(A)** A total of 95 patients were enrolled in the study. Patients had different T staging (T1, 8; T2, 36; T3, 31; T4, 20), lymph node involvement (N_0_, 42; N+, 53), and histological grading (well-differentiated, 26; moderately differentiated, 61; poorly differentiated, 3; others, 5). **(B)** Out of 95 patients, 71 had detectable circulating tumour cells (CTCs) at diagnosis. Those 71 CTC-positive patients had different T staging (T1, 7; T2, 27; T3, 23; T4, 14), lymph node involvement (N_0_, 32; N+, 39), and histological grading (well-differentiated, 20; moderately differentiated, 44; poorly differentiated, 2; others, 5).

**Figure 10:**
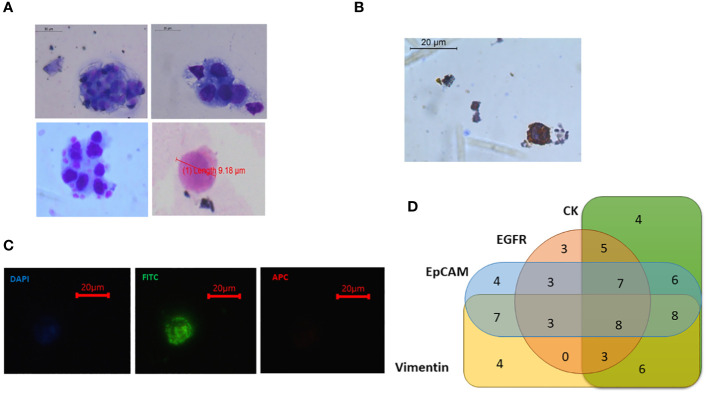
**(A)** Isolated potential circulating tumour cells (CTCs) from oral squamous cell carcinoma (OSCC) patients showing morphological characteristics of cancer cells [larger size >8 μm, high nuclear-to-cytoplasmic ratio, coarse nuclei, and irregular shape]. Some small-sized CTCs were also observed (<8 μm). **(B)** CTCs showing cytokeratin positivity. **(C)** Sorted CTCs were observed under fluorescence microscope. Fluorescein isothiocyanate (FITC)-positive, 4′,6-diamidino-2-phenylindole (DAPI) dim, and APC-negative cells were seen. **(D)** Out of 95 patients, 71 had detectable CTCs in their blood. Venn diagram showing that different CTC-positive samples had the expression of various CTC-specific markers (EpCAM, EGFR, CK-19, and Vimentin) in different combinations (**Table 1**).

Next, our technique was tested to isolate CTCs from the whole blood of 95 OSCC patients. Whole blood at a volume of 10 mL was collected from histologically proven OSCC patients, and CTCs were isolated using the standardised protocol (whole blood → PBMC isolation → CD45 depletion → detection and sorting of cells using optimised gating strategy). Nearly 75% (71/95) of patients had detectable CTCs in their blood (**Figure 9**). There was no difference in CTC positivity rate between early-stage (TNM I and II, 74.41%, n = 32/43) and late-stage (TNM III and IV, 75%, n = 39/52) patients. Sorted potential CTCs were further reconfirmed and characterised by various methods. Giemsa staining showed cancer cell-like morphological features, e.g., irregular shape, high nuclear-to-cytoplasmic ratio, and coarse chromatin (**Figure 10A**). Sorted potential CTCs were positive for cytokeratin as assessed by immunocytochemistry analysis. Whole transcriptome amplification followed by qRT-PCR for various CTC-specific genes (EpCAM, EGFR, CK-19, and Vimentin) revealed extensive phenotypic heterogeneity in potential CTCs isolated from OSCC patients using our technique (**Figure 10** and **Table 1**).

**Table 1:** Showing the different combinations of marker positivity in CTCs isolated from OSCC patients.

Marker positivity	Number of patients
All four markers	8
EpCAM + EGFR + CK	7
EpCAM + EGFR + Vimentin	3
EpCAM + CK + Vimentin	8
EGFR + CK + Vimentin	3
EpCAM + EGFR	3
EpCAM + CK	6
EpCAM + Vimentin	7
EGFR + Vimentin	0
EGFR + CK	5
CK + Vimentin	6
EpCAM	4
EGFR	3
CK	4
Vimentin	4

CTCs, circulating tumour cells; OSCC, oral squamous cell carcinoma.

### Isolated CTCs suitable for various downstream applications

**Figure 11:**
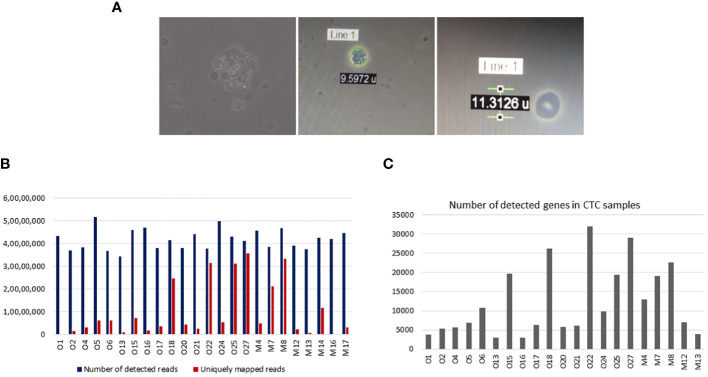
Downstream applications of isolated circulating tumour cells (CTCs). **(A)** Isolated CTCs were cultured for 4 weeks. **(B)** Ultra-low cell RNA sequencing of isolated CTCs was performed in 24 samples. Bar graphs show the number of detected and uniquely mapped reads for each 24 samples. The median detected reads were 41.7 million (range 34.4–51.8 million), and the median uniquely mapped reads were 4.7 (range 1.5–35.5 million). **(C)** Bar graphs showing the number of detected genes in CTC samples. The median detected genes were 7,099 (range 2,968–31,902).

We have already shown that isolated CTCs can be analysed by Giemsa staining, immunocytochemistry, and qRT-PCR analysis. Isolated CTCs were also cultured, and short-term CTC cultures were developed. These cells were successfully cultured for up to 4 weeks (**Figure 11A**). Isolated CTCs from 24 OSCC patients were also subjected to ultra-low cell RNA sequencing. The average, minimum, and maximum read depths obtained after demultiplexing and alignment were 41.7 million, 34.3 million, and 51.8 million, respectively. All the samples passed quality control (QC) before and after sequencing. Raw read counts, uniquely mapped read counts, and the number of detected genes are shown in **Figures 11B, C**.

## Discussion

Studying and establishing the evolution of solid cancers at the molecular level for clinical purposes remained limited, as obtaining tissue biopsies is invasive, technically challenging, and not always feasible at multiple sites and multiple time points (14). Moreover, tissue biopsies do not always reflect complex molecular features of solid tumours due to spatial and temporal heterogeneity within the solid tumour (15). On the contrary, analysis of CTCs provides a minimally invasive and easily repeatable approach for real-time monitoring of the disease process, which is essential for treatment success. As tumour evolves spatially and temporally (16), it is more important to analyse the molecular features of CTCs rather than their detection or enumeration (17). CTCs might represent the entire spectrum of clones in a solid tumour at a specific time, suggesting heterogeneity within the CTC population. Various studies suggested extensive phenotypic heterogeneity in CTCs (18–20).

We demonstrated a novel flow-based strategy to detect and isolate CTCs taking into account their heterogeneity. This strategy allows the detection and isolation of viable CTCs with varying surface markers, ready for downstream molecular analysis. FACS is widely used in detecting and isolating rare cells. This strategy is suitable for healthcare, as FACS is readily available, most healthcare institutions are well-equipped, and FACS can be operated with minimal technical expertise. In pre-enrichment steps, PBMCs are isolated from the whole blood using density gradient centrifugation followed by depletion of the CD45+ cell population. These pre-enrichment steps reduce the necessity to read millions of events in FACS and allow the isolation of pure CTCs without any contamination of background blood cells with better efficiency and specificity in less amount of time.

**Figure 12:**
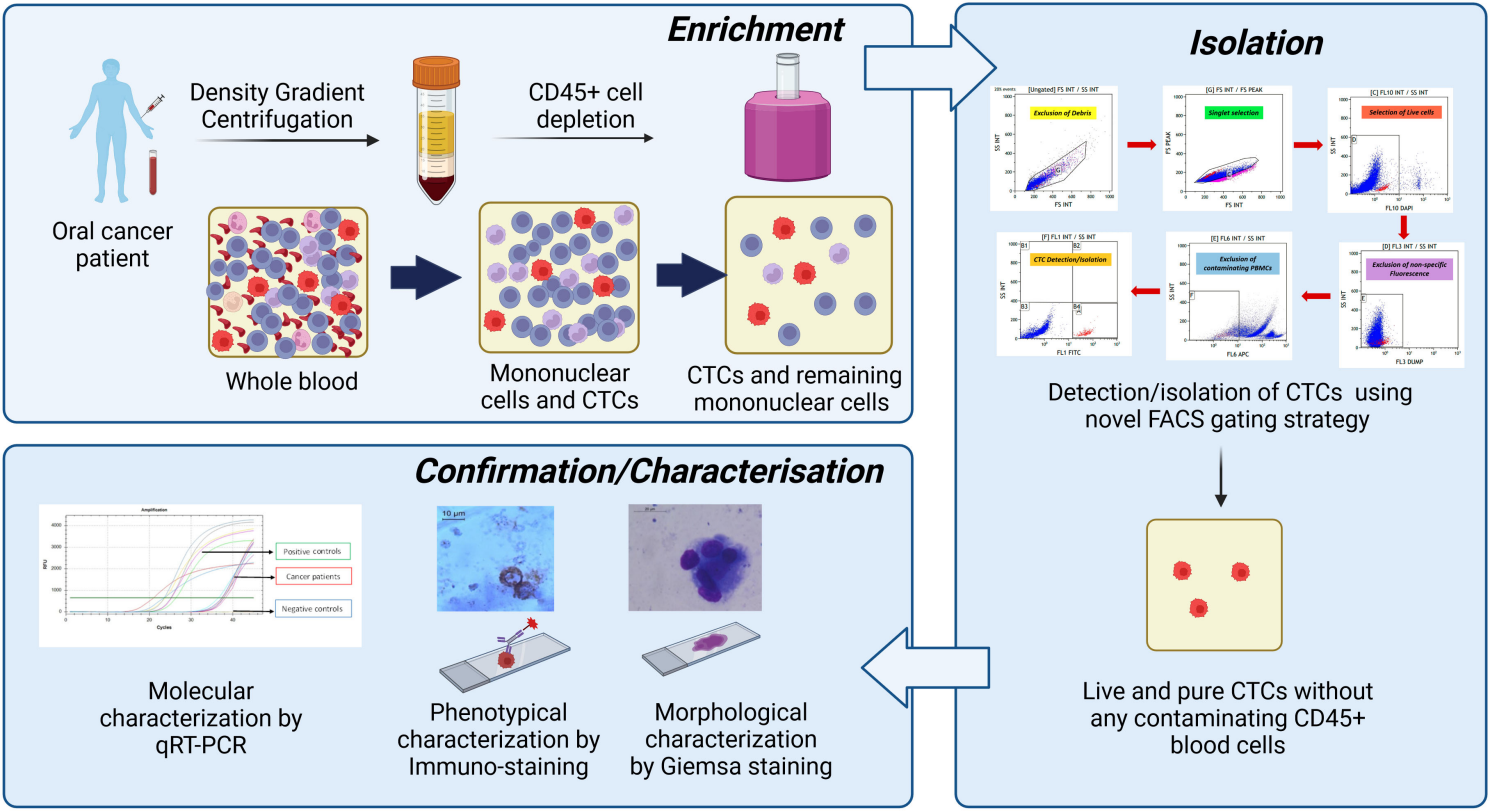
Summary of the developed technique. First, whole blood was subjected to peripheral blood mononuclear cell (PBMC) isolation followed by magnetic depletion of CD45+ hematogenous cells. The potential cancer cells were positively selected using developed novel fluorescence-activated cell sorting (FACS)-based techniques using the multi-marker approach. Then, isolated circulating tumour cells (CTCs) were reconfirmed/characterised by various methods like Giemsa staining (morphological characterisation), immuno-staining (phenotypic characterisation), and qRT-PCR (molecular characterisation).

Next, a stringent but efficient gating strategy was optimised to detect heterogeneous CTCs without requiring much technical expertise. Our gating strategy was able to strictly distinguish between cancer cells and blood cells, which is a crucial requirement for capturing rare cancer cells against the massive background of blood cells. Five exclusion gates were added to ensure the specificity of the gating. First, FSC and SSC discriminators were used to remove debris contamination. Then, doublet events were excluded using forward scatter peak (FS PEAK) versus forward scatter integral (FSC INT) dot plot. Next, DAPI was added, as it does not penetrate live cells in low concentrations; live cells were captured by gating DAPI versus a side scatter dot plot. Next, another exclusion gate, i.e., dump channel (Texas red), was added, as any conjugate was not specific to this channel; any positive signal corresponds to non-specific signals (21). This gate enabled the exclusion of events exhibiting any non-specific fluorescence. After this, CD45+ events (contaminating blood cells) were eliminated by plotting APC against the SCC discriminator. Finally, the CTC population was selected using a fascinating approach. FITC-labelled antibodies were used against four CTC-specific markers, EpCAM, EGFR, Pan CK (epithelial markers), and Vimentin (EMT marker), to capture CTCs with varying phenotypes. Using both epithelial and EMT markers for CTC selection provides the advantage of efficient CTC capture, as CTCs exhibit extensive phenotypic heterogeneity. Some CTCs are strictly epithelial or mesenchymal, while some are in varying degrees of transition phases of EMT (22–25). Studies have shown that CTCs with intermediate EMT phenotype are better metastasisers than purely with epithelial or mesenchymal phenotype (26, 27). Considering these facts, four CTC-specific markers were selected to detect the phenotypic heterogeneity of CTCs. The flow-sorted cells were reconfirmed and characterised using various methods like Giemsa staining (phenotypical characterisation), immuno-staining, and qRT-PCR (molecular characterisation) (**Figure 12**).

A pertinent point to note here is that the detection efficiency of our technique is comparable with that of some of the already established CTC isolation techniques (28). The detection efficiency of the Food and Drug Administration (FDA)-approved CellSearch™ system ranges from 42% to 90% (29–32), and for the Parsortix device, it ranges from 42% to 70% in HNSCC (33). It is worth mentioning that although our technique is not more efficient in enumerating the CTCs, it is better in identifying CTCs with broad size ranges and multiple markers. As a result, the CTC positivity rate in our patients is ~75%, which is higher as compared to that of other techniques. Other groups detected CTCs in 29%–77% of HNSCC patients using flow cytometry (6, 34–36). However, only 15%–40% of HNSCC patients had detectable CTCs when identified using CellSearch™ (6, 36–39), which counts CTCs strictly of epithelial origin. Currently, other available techniques explore either size-based approaches or approaches based on a single marker (majorly epithelial) to detect/isolate CTCs. Out of 95 patients, only 15 (15.7%) patients had CTCs positive for only a single marker, i.e., EpCAM (n = 4), EGFR (n = 3), CK (n = 4), and Vimentin (n = 4). Moreover, the addition of Vimentin as a selection marker for CTCs included the CTCs with EMT transformation, an important event associated with the metastatic process. Along with this, nearly 35% (25/71) of patients had EpCAM-negative CTCs, indicating the importance of detecting CTCs using a multi-marker approach. Our technique overcomes the disadvantages of currently available CTC isolation techniques, as it is independent of size-based bias. It isolates CTCs using a multi-marker-based approach, including EMT markers and epithelial markers, instead of a single-marker approach.

Another advantage of this FACS-based strategy is flexibility. Many more markers could be extended based on recent advances in literature and specific cancer types without requiring more fluorochromes (FITC-labelled antibodies against various markers could be added). This minimises the expertise needed to run various compensation controls for optimising multi-fluorochrome experiments and could make the strategy more reliable and sensitive.

Moreover, the strategy is able to isolate viable CTCs, which can be used for various downstream applications, like short-term CTC cultures, which further be used to develop CTC-derived xenograft models. The successful RNA sequencing (RNA-Seq) data, as shown by us, using ultra-low cell RNA-Seq strategy, successfully, may be used to analyse the transcriptomic signatures of CTCs as a temporal snapshot of tumour/CTC heterogeneity and to shed light on various biological aspects of the metastatic process.

Despite various advantages, we would like to discuss a few limitations of this study. First, we could not determine the exact CTC count in a particular sample because FITC-positive events in FACS do not necessarily reflect the actual number of CTCs. Second, we could not experimentally compare our technique with other well-known techniques like CellSearch™ because these techniques are not available in our country. As our technique is FACS-based, it is not suitable for the identification of homotropic and heterotrophic CTC clusters.

## Conclusion

In conclusion, here, we report an innovative CTC isolation technique that is sensitive, specific, flexible, and affordable, which overcomes the inherent limitations of currently available CTC detection techniques, provides a snapshot of CTC heterogeneity, and can isolate CTCs ready for downstream molecular analysis.

## Data availability statement

The original contributions presented in the study are included in the article/**Supplementary Material**. Further inquiries can be directed to the corresponding authors.

## Ethics statement

The studies involving humans were approved by Institute Ethics Committee, PGIMER, Chandigarh. The studies were conducted in accordance with the local legislation and institutional requirements. The participants provided their written informed consent to participate in this study.

## Author contributions

AC: Conceptualization, Data curation, Formal analysis, Investigation, Methodology, Validation, Writing – original draft. AP: Conceptualization, Data curation, Formal analysis, Investigation, Methodology, Writing – original draft, Funding acquisition, Project administration, Resources, Supervision, Writing – review & editing. MS: Investigation, Writing – review & editing. GB: Data curation, Writing – review & editing, Investigation, Methodology. MP: Writing – review & editing, Data curation. JB: Data curation, Writing – review & editing. RV: Data curation, Writing – review & editing. DC: Data curation, Writing – review & editing, Formal analysis, Investigation, Methodology, Writing – original draft. RS: Conceptualization, Data curation, Investigation, Methodology, Writing – review & editing. AM: Data curation, Writing – review & editing. SG: Data curation, Writing – review & editing, Conceptualization, Project administration.
