# Supplementary material for: A FACS-based novel isolation technique identifies heterogeneous CTCs in oral squamous cell carcinoma
Source: Front Oncol. 2024 Feb 26;14:1269211. doi: 10.3389/fonc.2024.1269211 (PMC10925612; doi:10.3389/fonc.2024.1269211)
Supplement: Supplementary file 1 [file DataSheet_1.docx]

| **Gene** | **Forward primer (5’-3')** | **Reverse primer (5’-3’)** |
| --- | --- | --- |
| EGFR | CTCAGCCACCCATATGTACC | GGTCTCGGGCCATTTTGG |
| EpCAM | AATGAATGGCTCAAAACTTGG | CAGTAGGTTCTCACTCGCTCAG |
| Cytokeratin 19 | GGTCAGTGTGGAGGTGGATT | TCAGTAACCTCGGACCTGCT |
| Vimentin | TCTACGAGGAGGAGATGCGG | GGTCAAGACGTGCCAGAGA |
| CD-45 | CTGACATCATCACCTAGCAG | TGCTGTAGTCAATCCAGTGG |
| 18S | GTAACCCGTTGAACCCCATT | CCATCCAATCGGTAGTAGCG |
| β-actin | CCCTATAAAACCCAGCGGC | AAGGTCTCAAACATGATCTGGGT |
| Puromycin resistance Gene | CCGAGTACAAGCCCACGG | AGAGTTCTTGCAGCTCGGTG |

**qRT-PCR conditions:**

1. **SYBR reaction**

95 °C – 2 min.

95 °C – 5 sec.

60 °C– 30 sec. 45 cycles

72°C – 30 sec.

Melt curve – 65 °C to 95 °C an increment of 0.5°C for every 5 sec**.**

1. **10uL Taqman Assays**

95 °C – 2 min.

95 °C – 5 sec.

60 °C– 30 sec. 45 cycles

**Formation of CRNN and puromycin-resistance gene expressing Cal 27 cell line:**

**Cell culture**

The Cal27 human tongue cancer cell line and HEK293T cells were gifts from Prof SS Chauhan. The cells were cultured at 37°C under 5% CO2 in Dulbecco's Modified Eagle Medium (DMEM) supplemented with 10% fetal bovine serum and 1% penicillin/streptomycin.

**Lentiviral plasmids:**

The pLenti CMV GFP Puro (658-5) (Addgene # 17448), psPAX2 (#12260 Addgene) and pMD2.G (#12259 Addgene) were used for the experiments. The Cornulin cloned into pcDNA3.1/V5-His TOPO TA vector was gifted by Chen et al. laboratory.

**Cloning of the human Cornulin coding sequence:**

The Cornulin coding sequence was amplified from pcDNA3.1/V5-His TOPO TA using primers as given in supplementary data. Cornulin expressing lentiviral plasmid was constructed by cloning the coding sequence of CRNN into the empty backbone of the pLenti CMV GFP Puro (658-5). The details for cloning are listed in Supplementary Data 1.

**Supplementary data 1:**

The Cornulin coding sequence was amplified using the primers listed below. These primers added BamH1 and Sal1 sites at the starting and end of the Cornulin amplified coding sequence (insert DNA). The amplified product was then restriction digested using BamH1 and Sal1, followed by PCR purification.

|  | **Primer sequence** |
| --- | --- |
| **CRNN Forward** | 5' TAGAGGATCCTCGCCACCATGGTGCCTCAGTTACTGCAAAACATTAATGG 3' (BamHI) |
| **CRNN Reverse** | 5’ GATTGTCGACGCGGCCGCTTCATGGCTTGGTGCTTCTCAAG 3’ (SalI) |

The pLenti CMV GFP Puro (658-5) plasmid was restriction digested using BamH1 and Sal1. The backbone of the plasmid (~7.9kb) was then gel extracted. The ligation was performed using T4 DNA Ligase (# EL0012 Thermo Scientific) in a 5:1 molar ratio of Insert DNA: Vector DNA. The 5ul of ligation mixture was used for the transformation of 50ul of the chemically competent Stbl3. The inserted clones were selected with ampicillin and confirmed for the CRNN gene by colony PCR.

**Transfection of HEK293T cells and Production of Lentiviral titre:**

HEK293T was seeded at the density of 0.5×10^6^ cells/well. When the cells reached about 70-80% confluency were transfected with the recombinant plasmid (CRNN inserted) 500ng, along with the two other plasmids i.e. psPAX2 375ng and pMD2.G 125ng using Lipofectamine 3000 (# L3000008 Invitrogen; Thermo Fisher Scientific) according to manufacturers' protocol. The lentiviral supernatants were harvested at 24hours and 48hours, followed by centrifugation at 500 × g for 10 min. The viral supernatant was filtered through a 0.45 μm filter and stored at − 80 °C for storage.

**Transduction of Cal27 cell line:**

Cal27 cells were seeded in the 6 well plates at the density of 0.5×10^6^ cells/well. At 70-80% confluency, the cells were transduced with the lentiviruses in a 1:1 ratio of viral titre: complete media with 8 μg/mL polybrene. Transduced cells were then selected with puromycin for 7-10 days using the concentration based on the killing curves. The selected cells were then subjected to single colony screening.
